# Dr. Charles Forbes

**Published:** 1917-12

**Authors:** 


**Dr. Charles Forbes,** P. & S. (Columbia) 1871, of Rochester, died in the Rochester General Hospital, Oct. 2, aged 73. He was a scientist, especially interested in electricity and photography. To him is due the individual communion cup and several minor medical appliances.

